# Just the Facts: Protected code blue – Cardiopulmonary resuscitation in the emergency department during the coronavirus disease 2019 pandemic

**DOI:** 10.1017/cem.2020.379

**Published:** 2020-04-24

**Authors:** Sarah McIsaac, Randy S. Wax, Brit Long, Christopher Hicks, Christian Vaillancourt, Robert Ohle, Paul Atkinson

**Affiliations:** * Division of Critical Care, Department of Medicine, Northern Ontario School of Medicine, Sudbury, ON; † Health Science North Research Institute, Sudbury, ON; ‡ Department of Critical Care Medicine, Lakeridge Health, Oshawa, ON; § Department of Critical Care Medicine, Faculty of Health Sciences, Queen's University, Kingston, ON; ¶ Department of Emergency Medicine, SAUSHEC, Fort Sam Houston, TX; ** Division of Emergency Medicine, Department of Medicine, University of Toronto, Toronto, ON; †† Department of Emergency Medicine, Ottawa Hospital Research Institute, University of Ottawa, Ottawa, ON; ‡‡ Department of Emergency Medicine, Northern Ontario School of Medicine, Sudbury, ON; §§ Dalhousie University, Saint John Regional Hospital, Saint John, NB

**Keywords:** Cardiac arrest, COVID-19, protected code blue

## Abstract

Emergency medical services (EMS) is called for a 65-year-old man with a 1-week history of cough, fever, and mild shortness of breath now reporting chest pain. Vitals on scene were HR 110, BP 135/90, SpO2 88% on room air. EMS arrives at the emergency department (ED). As the patient is moved to a negative pressure room, he becomes unresponsive with no palpable pulse. What next steps should be discussed in order to protect the team and achieve the best possible patient outcome?

## CLINICAL SCENARIO

Emergency medical services (EMS) is called for a 65-year-old man with a 1-week history of cough, fever, and mild shortness of breath now reporting chest pain. Vitals on scene were HR 110, BP 135/90, SpO2 88% on room air. EMS arrives at the emergency department (ED). As the patient is moved to a negative pressure room, he becomes unresponsive with no palpable pulse. What next steps should be discussed in order to protect the team and achieve the best possible patient outcome?

## KEY CLINICAL QUESTIONS

1.: **What is a protected code blue (PCB)?**

*Answer:* A PCB is an emergency response to a life-threatening illness in a patient with a suspected or confirmed novel respiratory or communicable illness. The PCB concept was developed during the severe acute respiratory syndrome coronavirus (SARS-CoV) crisis in 2003, when many healthcare providers were exposed to SARS-CoV, resulting in several deaths. The PCB shifts the mentality away from patient outcome and puts healthcare provider safety front and centre.^1^ The decision to initiate a PCB is based on suspected or confirmed presence of a novel respiratory illness. If a reliable history of this cannot be obtained, PCB should be initiated. In the setting of significant community burden of disease, the decision to treat all cardiac arrests as protected should be considered.

2.: **Is cardiopulmonary resuscitation (CPR) an aerosolizing procedure?**

*Answer:* CPR refers to the combination of chest compressions, defibrillation, and airway management. The World Health Organization and the American Heart Association list CPR as an aerosolizing medical procedure.^2,3^

There are no high-quality studies to reliably answer this question. A meta-analysis published in 2013 demonstrated that intubation was associated with increased healthcare provider infection.^4^ For chest compressions, three retrospective studies of low quality (Liu et al. 2009, Loeb et al. 2004, and Raboud et al. 2010) were included. Only one study found an increased odds of infection (Liu et al. 2009), but chest compressions were performed in conjunction with intubation; thus, it is unclear whether the increased risk of infection is attributable to performing chest compressions, airway management, or a combination of the two.^4^ Defibrillation alone was not associated with increased risk of healthcare provider infection.^4^

3.: **What personal protective equipment (PPE) is recommended during a PCB?**

*Answer:* General recommendations from leading resuscitation experts include a fluid-resistant gown, long-sleeved gloves, full face shield with eye protection, and fit-tested N95 respirators. Hair covers or hoods should also be worn.

Defibrillation is *not considered* to be an aerosolizing procedure; therefore, contact droplet precautions are sufficient. However, given the potential association of chest compressions with aerosolization, enhanced airborne precautions should be worn by all team members *prior* to commencing *any other* resuscitative efforts. The resuscitation should take place in a negative pressure room if one is available. Powered air purifying respirators are not readily available and have not been shown to reduce viral transmission.^5^ ED and EMS providers should have PPE, including airborne precautions (i.e., N95), readily available at all times given the risk of coronavirus disease 2019 (COVID-19) infection in the undifferentiated cardiac arrest.

In making PPE recommendations, we acknowledge the relative lack of high-quality available data from which current public health policy is derived evaluating the likelihood of aerosol production from chest compressions. It is therefore acceptable for a staff member to don droplet contact or full enhanced airborne PPE, including an N95 respirator.

4.: **What team members form the PCB team?**

*Answer:* The guiding principle for a PCB is to minimize the number of people in the room. A team leader plus three additional members represent a reasonable trade-off between team size and coordination of multiple tasks.

Minimizing the number of individuals in the room reduces the risk of exposure. The exact number of people will vary by institution culture and availability of personnel. A code team leader (physician or nurse) plus two CPR-trained support staff of any designation (to cycle and perform chest compressions) would be sufficient for the initial stages of the PCB; however, four team members would allow regular cycles between individuals performing chest compression and a dedicated airway leader (physician). When resources permit, a designated safety and logistics officer who remains *outside* of the room can observe strict adherence to team safety and donning and doffing of PPE. An additional member outside of the room in enhanced airborne precautions PPE may allow for a quick relief of chest compression duties.

5.: **When should I intubate the patient in a PCB?**

*Answer:* Early intubation should be prioritized to reduce aerosolization. Chest compressions should not be delayed for intubation but must be paused during intubation to facilitate first-pass success and reduce risk of viral droplet/aerosol spread. If the patient is making any respiratory effort, rapid sequence intubation medications should be provided prior to attempting intubation.

**Figure 1.**
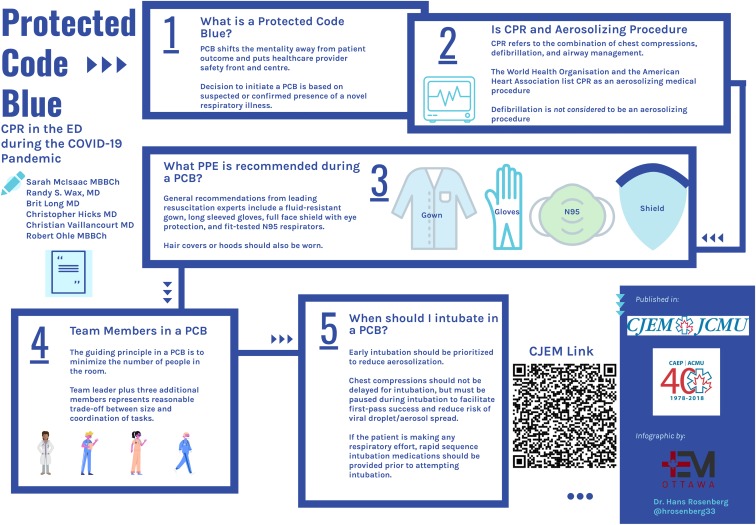
Inforgraphic depicting Protected Code Blue.

In order to protect the PCB team, intubation should be prioritized to establish closed circuit ventilation. A fluid resident face mask should be placed on the patient's mask to shield the team from any droplets. A high flow non-rebreather O2 mask with a HEPA filter can be considered to enable passive oxygenation; however, care should be taken to turn off the O2 flow prior to intubation. Bag-valve-mask ventilation is problematic in a PCB, as it is an aerosolizing procedure, and it can be difficult to maintain an adequate seal during a cardiac arrest. Intubation should be performed by the most experienced operator optimized for first-pass success. Video laryngoscopy is suggested as the preferred modality, as it allows the operator to increase distance from the patient's head. Chest compressions should be held while intubation is performed until the endotracheal tube placement is confirmed and closed circuit is established. Insertion of a supraglottic airway with an attached filter is preferred to bag valve mask ventilation as a rescue manoeuvre in the event of a difficult intubation.

## CASE RESOLUTION

Outside of the room, the PCB team dons enhanced airborne PPE under supervision of a safety officer. Inside of the room, a fluid resistant mask is applied to the patient, and the patient is quickly transferred to the ED stretcher. A nurse in droplet contact precautions attaches the patient to a defibrillator. The patient is in ventricular fibrillation and defibrillation is provided.

The PCB team enters, after the nurse in contact droplet leaves chest compressions are commenced, until the team is ready to intubate. The physician states loudly “ready to intubate,” and the nonessential team steps away from the patient while chest compressions are held. The airway is secured quickly with a video laryngoscope; no ventilation is performed until the airway is secured and the endotracheal cuff is inflated.

After a further 2 minutes, return of spontaneous circulation is achieved. The patient is stabilized in the ED and transferred according to local infection prevention and control protocols. The team doffs safely under supervision of a safety officer and debriefs.

**KEY POINTS**

• Early defibrillation can be provided by staff wearing only contact/droplet precautions prior to entry of the PCB team.
• All PCB team members should be in appropriate enhanced airborne precautions *before* entering the room and commencing higher risk resuscitation interventions.
• No bag-valve-mask ventilations are to be performed prior to tube delivery and cuff inflation.
• Early intubation should be performed by the most experienced provider and chest compressions paused to facilitate first-pass success.
• A safety officer is recommended to ensure strict adherence to safe donning and doffing.
